# Toxicity, baseline of susceptibility, detoxifying mechanism and sublethal effects of chlorogenic acid, a potential botanical insecticide, on *Bemisia tabaci*

**DOI:** 10.3389/fpls.2023.1150853

**Published:** 2023-02-22

**Authors:** Ran Wang, Qinghe Zhang, Cheng Qu, Qian Wang, Jinda Wang, Chen Luo

**Affiliations:** ^1^ Institute of Plant Protection, Beijing Academy of Agriculture and Forestry Sciences, Beijing, China; ^2^ National Engineering Research Center for Sugarcane, Fujian Agricultural and Forestry University, Fuzhou, China

**Keywords:** *Bemisia tabaci*, chlorogenic acid, botanical insecticide, metabolic enzymes, cytochrome P450 monooxygenases, sublethal effects

## Abstract

*Bemisia tabaci* is a threat to agriculture worldwide because of its potential to cause devastating damage to crops. Chlorogenic acid is a bioactive pesticidal phytochemical agent against various insect pests. We here determined the susceptibility of a laboratory strain of *B. tabaci* to chlorogenic acid and other popular insecticides, and the susceptibility of several field-collected populations to chlorogenic acid. Also, cross-resistance to four common insecticides was measured. Chlorogenic acid had the highest toxicity of all tested insecticides, and all the field-collected populations were susceptible to chlorogenic acid, and little cross-resistance was detected between chlorogenic acid and the other tested insecticides. Furthermore, analysis of enzyme activities and expression of P450 genes in *B. tabaci* after treatment with LC_50_ of chlorogenic acid suggested that enhanced P450 activity could be involved in chlorogenic acid detoxification. We subsequently evaluated sublethal effects of chlorogenic acid, and found that treatment with LC_25_ of chlorogenic acid prolonged duration of two developmental stages, reduced fecundity, and decreased survival rates of treated *B. tabaci* compared to untreated insects. Overall, these findings demonstrate strong toxicity and significant sublethal effects of chlorogenic acid on *B. tabaci*, and suggest that overexpression of P450 genes may be associated with chlorogenic acid detoxification.

## 1 Introduction

Pest management is a necessary aspect of agricultural production. Chemical insecticides are a major pest control measure, and have thus been extensively applied against insect pests for decades, generally with high efficacy. However, this long-term application of chemical insecticides has had detrimental side effects, such as sublethal effects on non-target insects, high levels of chemical residues in the environment and the food web, and ecosystem destruction (Sharma et al., 2020). Bioinsecticides have been suggested as appropriate alternatives to chemical agents owing to their decreased toxicity, high biodegradability, excellent target specificity, and minimal adverse effects on non-target organisms (Wang S. et al., 2022). Plants have developed many environmental adaptations, including physiological alterations, to cope with herbivore attacks. Specialized metabolites are natural plant products that play important roles in safeguarding plants against insect pests; some such compounds have been screened for their potential as commercial pest management products (Divekar et al., 2022). For example, the alkaloid compound caffeine has insecticidal properties; it causes paralysis and intoxication by inhibiting herbivore phosphodiesterase activity, and is therefore regarded as a potential biopesticide (Hollingsworth et al., 2002). Development of botanically-derived pesticides may be a feasible and environmentally sustainable strategy of preventing insect damage to crops.

Several key types of phytochemicals, such as flavonoids and phenolics, have important functions in herbivore resistance (Yao et al., 2019; Xia et al., 2021). Plant phenolic metabolites including chlorogenic acid, tannic acid, and methyl jasmonate show toxicity against insect pests, adversely affecting key physiological processes (Kundu and Vadassery, 2019; Lin et al., 2021; Lin et al., 2022). Chlorogenic acid is reportedly associated with the phytochemical defenses of plants such as *Dendranthema grandiflora* and *Ipomoea batatas* against insect pests such as *Frankliniella occidentalis* and *Cylas formicarius*, respectively (Leiss et al., 2009; Liao et al., 2020). Recently, Wang et al. (2021) reported that chlorogenic acid content was greatly increased as a result of *Mythimna separate* feeding, and that chlorogenic acid displayed significant toxicity against *M*. *separate* larvae. In recent years, plant-derived pesticidal compounds have become a focus of research attention due to their safety and lack of general environmental toxicity. Chlorogenic acid is one potential botanical insecticide that is highly environmentally friendly compared to common synthetic insecticides.

Insect oxidase systems include cytochrome P450 monooxygenases (P450s), which are multifunctional biocatalysts with broad enzymatic activity on a variety of substrates. Metabolic detoxification is one of the common mechanisms of resistance to various xenobiotics, and P450s are critical in the detoxification of natural and synthetic toxins (Lu et al., 2020; Nauen et al., 2021). Insect exposure to xenobiotics such as pesticides and plant specialized metabolites can induce high expression of P450 genes; for example, in cotton bollworm, coumarin treatment up-regulates the P450 genes *CYP6B7*, *CYP6B6*, and *CYP6B2*, and decreases bollworm susceptibility to methomyl (Chen et al., 2018). Similarly, in *Spodoptera exigua*, gossypol treatment induces high expression of *CYP9A98* and *CYP6AB14*. It was recently reported that several concentrations of chlorogenic acid can induce expression of P450 genes in *M*. *separate*, and that three P450 genes in particular (*CYP321A7*, *CYP6B6*, and *CYP6B7-like*) may be responsible for detoxifying chlorogenic acid (Lin et al., 2022).

The whitefly *Bemisia tabaci* (Gennadius) is an agriculturally devastating insect pest with high genetic diversity that is distributed worldwide. It has been known to infest more than 600 host plant species, primarily feeding on the phloem (Wang et al., 2017). *B. tabaci* damages plants not only directly but also indirectly; it is capable of transmitting more than 100 different plant viruses through feeding (Wei et al., 2017). Extensive and long-term employment of various synthetic pesticides to control *B. tabaci* worldwide has led to increasing reports of insect resistance to these pesticides (Horowitz et al., 2020); it is thus urgent to identify an alternative that can be used to delay the development of insecticide resistance in an environmentally-friendly manner. In the present study, we confirmed the toxicity of chlorogenic acid in *B. tabaci*, determined the baseline susceptibility of field-sampled *B. tabaci* populations to chlorogenic acid and other pesticides, and assessed pesticide cross-resistance. We found that all field-sampled populations were highly susceptible to chlorogenic acid, and no cross-resistance to the other tested pesticides was observed. We then illustrated the biochemical mechanism of chlorogenic acid action by measuring the activities of glutathione S-transferase (GST), esterase (EST), and P450, and assayed the expression of related genes. Finally, we assessed the sublethal effects of chlorogenic acid on *B. tabaci*. In summary, this study describes the optimal use of chlorogenic acid against *B. tabaci* and lays the foundation for future research and development of chlorogenic acid as a novel botanical pesticide.

## 2 Materials and methods

### 2.1 Insects

*B. tabaci* strain MED-S was originally collected from damaged poinsettia plants (*Euphorbia pulcherrima* Wild. ex Klotz.) in Beijing, China in 2009 (Pan et al., 2011). Four populations of *B. tabaci* that were previously reported to be insecticide-resistant were tested for cross-resistance; these were an abamectin-resistant strain (XZ), an afidopyropen-resistant strain (HD-Afi), a cyantraniliprole-resistant strain (CYAN-R), and a flupyradifurone-resistant strain (FLU-SEL) (Wang et al., 2020a; Wang et al., 2020b; Wang et al., 2022b; Wang et al., 2022c). Populations of *B. tabaci* were collected from six Chinese provinces and tested for baseline susceptibility as previously described (Wang et al., 2022b). All field-collected populations were identified as Mediterranean (MED) cryptic species using a previously published method (Luo et al., 2002). Insects were initially fed on cotton plants *Gossypium hirsutum* (without pesticide exposure) under a 14/10 h light/dark photoperiod at 26 ± 2°C and 55 ± 5% relative humidity (RH). For all assays, adults that were 3 d old or younger were sampled at random; approximately equal numbers of male and female individuals were used.

### 2.2 Insecticides

All chemical agents tested were of analytical standard grade. Chlorogenic acid (Chemical Abstracts Service [CAS] #327-97-9), abamectin (CAS #71751-41-2), flupyradifurone (CAS #951659-40-8), cyantraniliprole (CAS #736994-63-1), imidacloprid (CAS #138261-41-3), thiamethoxam (CAS #153719-23-4), flonicamid (CAS #158062-67-0), acetamiprid (CAS #160430-64-8), clothianidin (CAS #210880-92-5), nitenpyram (CAS #150824-47-8), and dinotefuran (CAS #165252-70-0) were purchased from Sigma Aldrich (Shanghai, China). Afidopyropen (CAS #915972-17-7) and sulfoxaflor (CAS #946578-00-3) were purchased from Dr. Ehrenstorfer (Augsburg, Germany).

### 2.3 Toxicity of chlorogenic acid to *B. tabaci*

All bioassays were carried out on adult *B. tabaci* individuals using an artificial diet solution as described by Wang et al. (2023). Five separate working concentrations were made for each chemical agent with four replicates per concentration. Thirty to forty *B. tabaci* adults were sampled at random and introduced into a bioassay tube containing insecticide or artificial diet solution without any insecticides (the control), which constituted one replicate. After 96 h in the tube, *B. tabaci* were considered to be dead if they did not move even when touched with a fine-hair brush. Survival and death rates were then calculated and recorded.

### 2.4 Detoxifying enzyme gene expression and activity assays

Activities of three detoxifying enzymes (GST, EST, and P450) were measured as previously described (Wang et al., 2020a) with slight alterations. The median lethal concentration (LC_50_) treatment comprised adults that survived treatment with the LC_50_ concentration for 96 h, and the control group was made of insects treated with the control for the same period of time. For each group, 200 mixed-sex *B. tabaci* individuals were sampled as one replicate. Three replicates were sampled for each of the three detoxifying enzymes. Protein content was measured using bovine serum albumin (BSA) as the standard with the method described by Bradford (1976). Based on previous publications regarding P450-mediated pesticide resistance in *B. tabaci* (Wang Q. et al., 2020; Zhou et al., 2020), expression levels of 12 detoxifying genes were measured *via* quantitative reverse transcription (qRT)-PCR as previously described (Wang et al., 2020a): CYP6CX1v1, CYP6CX3, CYP6CX4, CYP6CX5, CYP6CM1, CYP6DW2, CYP6DW3, CYP6DZ4, CYP6DZ7, CYP303A1, CYP4C64, and CYP4G68. Expression data were normalized using *TUB1α* and *EF-1α* as the internal control genes, and the results were conducted in terms of the 2^−△△Ct^ method (Pfaffl, 2001). Primer sequences are shown in **Supplementary Table S1**.

### 2.5 Sublethal effects of chlorogenic acid on *B. tabaci*

The 25% lethal concentration (LC_25_) of chlorogenic acid was calculated based on the results of the assay described in Section 2.3. Several fitness parameters were then measured in *B. tabaci* in control and LC_25_-treated groups. The experiments were carried out as previously described (Wang et al., 2020a) with slight alterations. Briefly, 12 clean cotton plants were evenly divided between two separate insect-proof cages (one control [CK] cage and one LC_25_-treatment cage). After 96h feeding with LC_25_ or the control by the method of the bioassay, 120 adults of *B. tabaci* that were treated (LC_25_) were then moved into the LC_25_ cage for egg laying measurements, and 120 untreated *B. tabaci* adults were moved into the CK cage as the control group. After 12 h of oviposition, all the plants were moved out of the two cages, and 10 leaves were recorded from each of the cages. In each of the 20 leaves, 10 eggs were left on each leaf and kept with one leaf clip-cage. All the spots of the eggs on the working leaves were marked, and the cages were placed in the chamber with the room temperature. Newly emerged adults of *B. tabaci* were put onto new leaves with clip cages for fecundity measurements that continued until all tested ones died, and after that the hatch rate of eggs was recorded.

### 2.6 Statistical analysis

Probit analysis was conducted in PoloPlus (2002) to confirm the significance of the death rate statistics for insects exposed to the series of working concentrations of chlorogenic acid. Resistance ratio (RR) was calculated as LC_50_ (field-collected population)/LC_50_ (MED-S), and levels of pesticide resistance is reported by the published method (Zhang et al., 2022). Specifically, susceptibility with the RR less than 5-fold, low level of resistance with RR from 5- to 10-fold, moderate level of resistance with RR from 10- to 40-fold, high level of resistance with RR from 40- to 160-fold, and very high level of resistance with RR over 160-fold. Significant differences in *B. tabaci* growth duration, viability, fecundity, duration of oviposition, and egg hatchability between the CK and treatment groups were assessed using Student’s *t*-test. Differences in detoxifying enzyme activity and gene expression were also assessed with Student’s *t*-test. All statistical analyses were conducted using SPSS (2011).

## 3 Results

### 3.1 Lethal effects of chlorogenic acid and popular insecticides on *B. tabaci*

The toxicity of chlorogenic acid and 11 other popular chemical agents were confirmed in the susceptible MED-S strain of *B. tabaci* using a feeding method as previously published (Wang et al., 2023) (**Table 1**). The death rate of the control group was < 5%. The chemical agent with the highest lethal effect against *B. tabaci* adults was chlorogenic acid (LC_50_ = 0.930 mg/L), followed by cyantraniliprole (LC_50_ = 1.347 mg/L) and flonicamid (LC_50_ = 1.398 mg/L), which also showed excellent toxicity against *B. tabaci*. The other chemical agents had significantly lower toxicity than chlorogenic acid: 3.5 times lower for dinotefuran (LC_50_ = 3.259 mg/L), 3.9 times lower for clothianidin (LC_50_ = 3.656 mg/L), 4.6 times lower for acetamiprid (LC_50_ = 4.299 mg/L), 6.0 times lower for nitenpyram (LC_50_ = 5.574 mg/L), 8.2 times lower for afidopyropen (LC_50_ = 7.617 mg/L), 10.7 times lower for sulfoxaflor (LC_50_ = 9.950 mg/L), 11.3 times lower for flupyradifurone (LC_50_ = 10.495 mg/L), 12.3 times lower for thiamethoxam (LC_50_ = 11.449 mg/L), and 23.1 times lower for imidacloprid (LC_50_ = 21.489 mg/L).

**Table 1:** Median lethal concentration (LC_50_) of chlorogenic acid and 11 popular insecticides on *Bemisia tabaci*.

Population	N ^a^	LC_50_ (95% CL) (mg L^-1^) ^b^	Slope ± SE	*X* ^2^ (df)
Chlorogenic acid	614	0.930 (0.656 - 1.200)	1.134 ± 0.135	2.542 (3)
Cyantraniliprole	615	1.347 (1.083 - 1.631)	1.281 ± 0.132	1.036 (3)
Flupyradifurone	617	10.495 (8.248 - 12.794)	1.297 ± 0.135	2.758 (3)
Imidacloprid	621	21.489 (16.285 - 26.696)	1.261 ± 0.134	1.492 (3)
Thiamethoxam	619	11.449 (9.160 - 13.822)	1.347 ± 0.134	1.942 (3)
Sulfoxaflor	626	9.950 (8.373 - 11.815)	1.425 ± 0.131	2.534 (3)
Afidopyropen	612	7.617 (6.095 - 9.164)	1.430 ± 0.140	2.296 (3)
Flonicamid	610	1.398 (1.080 - 1.728)	1.188 ± 0.131	1.605 (3)
Acetamiprid	611	4.299 (3.425 - 5.322)	1.122 ± 0.128	1.188 (3)
Clothianidin	624	3.656 (2.730 - 4.728)	1.596 ± 0.137	3.130 (3)
Nitenpyram	611	5.574 (4.317 - 6.876)	1.203 ± 0.132	1.410 (3)
Dinotefuran	607	3.259 (2.590 - 4.310)	1.067 ± 0.129	1.319 (3)

^a^Number of insects used. ^b^ CL, confidence limit.

### 3.2 Baseline susceptibility of field-collected *B. tabaci* to chlorogenic acid and other pesticides

**Figure 1:**
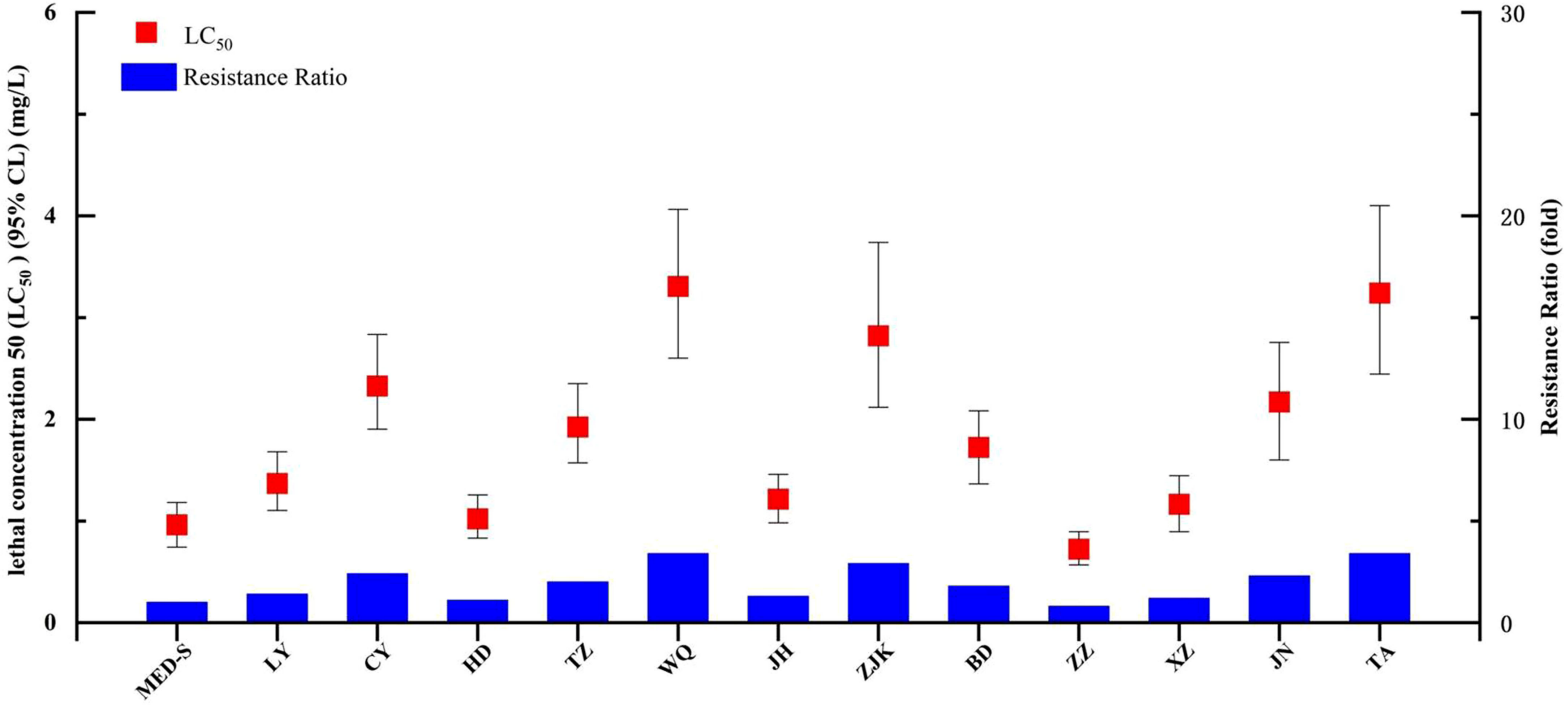
Susceptibility of *Bemisia tabaci* populations collected from fields in China to chlorogenic acid.

We next tested the baseline chlorogenic acid susceptibility of 12 *B. tabaci* MED populations collected in the field and one laboratory-maintained susceptible strain (MED-S) (**Figure 1** and **Table S2**). Of the field-collected strains, ZZ showed the highest susceptibility to chlorogenic acid (LC_50_ = 0.723 mg/L), whereas the MED-S strain displayed the highest susceptibility overall (LC_50_ = 0.962 mg/L). WQ had the lowest susceptibility to chlorogenic acid (LC_50_ = 3.306 mg/L), followed by TA (LC_50_ = 3.241 mg/L). The resistance ratios of all field-collected strains were less than five-fold different than that of the MED-S strain, indicating a lack of chlorogenic acid resistance in field populations. XZ was confirmed as an abamectin-resistant strain (41.6-fold resistance), HD-Afi was afidopyropen-resistant (174.9-fold resistance), CYAN-R was cyantraniliprole-resistant (99.3-fold resistance), and Flu-R was flupyradifurone-resistant (160.4-fold resistance). However, none of these strains showed chlorogenic acid resistance, suggesting that chlorogenic acid displayed little cross-resistance with these four other pesticides (**Table 2**).

**Table 2:** Cross-resistance of *Bemisia tabaci* against chlorogenic acid and four popular insecticides.

Insecticide	Strain	N ^a^	LC_50_ (95% CL) (mg/L) ^b^	Slope ± SE	*χ*2 (*df*)	RR ^c^
Chlorogenic acid	MED-S	615	0.888 (0.663 - 1.114)	1.192 ± 0.133	0.827 (3)	
	XZ	615	1.573 (1.156 - 1.986)	1.218 ± 0.136	1.204 (3)	1.8
	HD-Afi	630	1.723 (1.219 - 2.218)	1.172 ± 0.135	1.823 (3)	1.9
	CYAN-R	619	1.011 (0.753 - 1.266)	1.257 ± 0.136	1.211 (3)	1.1
	FLU-SEL	611	2.381 (1.838 - 2.951)	1.155 ± 0.130	0.775 (3)	2.7
Abamectin	MED-S	622	0.10 (0.070 - 0.120)	1.427 ± 0.169	2.701 (3)	
	XZ	615	4.159 (3.130 - 5.180)	1.274 ± 0.137	0.950 (3)	41.6
Afidopyropen	MED-S	617	5.581 (4.140 - 7.075)	1.043 ± 0.128	1.007 (3)	
	HD-Afi	632	976.163 (779.027 - 1221.122)	1.067 ± 0.125	1.289 (3)	174.9
Cyantraniliprole	MED-S	610	1.071 (0.837 - 1.312)	1.266 ± 0.134	1.581 (3)	
	CYAN-R	609	106.402 (83.674 - 129.673)	1.311 ± 0.135	2.828 (3)	99.3
Flupyradifurone	MED-S	616	9.969 (6.770 - 13.134)	1.001 ± 0.130	2.946 (3)	
	FLU-SEL	623	1599.386 (1250.543 - 1957.451)	1.265 ± 0.132	1.140 (3)	160.4

^a^Number of insects used. ^b^ CL, confidence limit. ^c^ Resistance ratio (RR) = LC_50_(strain XZ, HD-Afi, CYAN-R, or FLU-SEL)/LC_50_(strain MED-S).

### 3.3 Biochemical mechanism of *B. tabaci* response to chlorogenic acid treatment

**Figure 2:**
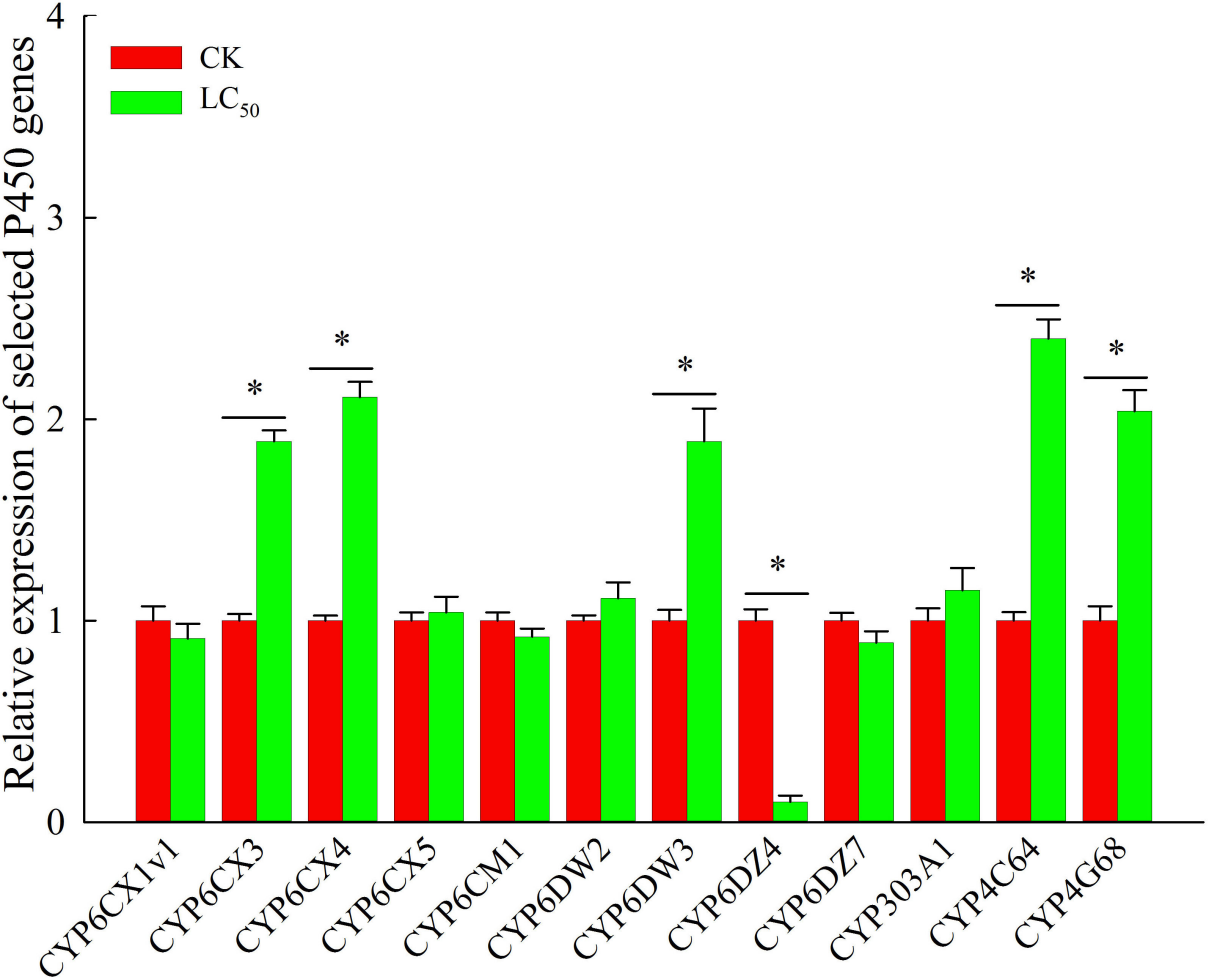
Expression profiles of 12 cytochrome P450 genes that may be involved in chlorogenic acid detoxification in *Bemisia tabaci* adults. Red, control (CK) individuals. Green, individuals treated with the median lethal concentration (LC_50_) of chlorogenic acid. Values are presented as the mean ± standard error. *p < 0.05 (Student’s *t*-test).

Chlorogenic acid-treated and control *B. tabaci* were assayed to measure the activity of three detoxifying enzymes: P450, GST, and EST. Compared with the control group, P450 activity was significantly elevated (by 1.9-fold) in the group treated with the LC_50_ concentration of chlorogenic acid; GST and EST activities were increased compared to the control group by 1.3-fold and 1.1-fold, respectively, but the differences were not significant (**Table 3**). In the control and LC_50_ groups, expression patterns were also analyzed *via* qRT-PCR for 12 P450 genes that have previously been reported as involved in detoxification: CYP6CX1v1, CYP6CX3, CYP6CX4, CYP6CX5, CYP6CM1, CYP6DW2, CYP6DW3, CYP6DZ4, CYP6DZ7, CYP303A1, CYP4C64, and CYP4G68. In comparison to the control group, *CYP6CX3*, *CYP6CX4*, *CYP6DW3*, *CYP4C64*, and *CYP4G68* were significantly up-regulated in the treated insects by 1.9-fold, 2.1-fold, 1.9-fold, 2.4-fold, and 2.0-fold, respectively. In contrast, *CYP6DZ4* was significantly down-regulated (by 10.0-fold) in the LC_50_-treated group (**Figure 2**).

**Table 3:** Metabolic enzyme activity in control (CK) *Bemisis tabaci* individuals and those treated with the median lethal concentration (LC_50_) of chlorogenic acid.^a^.

Treatment	P450s activity		ESTs activity		GSTs activity	
	pmol min^-1^ mg^-1^	Ratio ^b^	nmol min^-1^ mg^-1^	Ratio ^b^	nmol min^-1^ mg^-1^	Ratio ^b^
CK	0.68 ± 0.16		311.06 ± 18.93		40.26 ± 10.31	
LC_50_	1. 32 ± 0.24 *	1.9	329.75 ± 25.32	1.1	51.07 ± 11.12	1.3

^a^Mean activity values in a single column followed by an asterisk are significantly different (p < 0.05). ^b^ Ratio = activity in individuals treated with the LC_50_ dose/activity in CK individuals.

### 3.4 Sublethal effects of chlorogenic acid on *B. tabaci*

**Figure 3:**
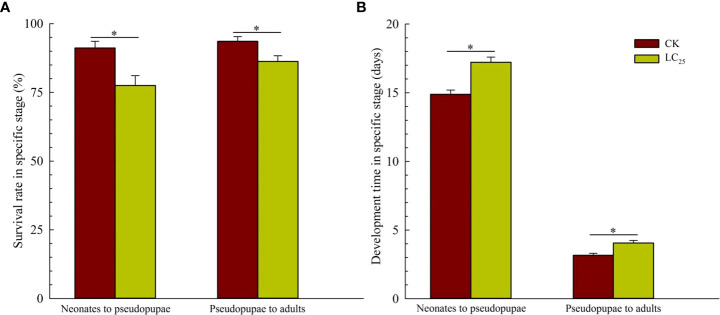
Survival rates **(A)** and developmental stage durations **(B)** in the F_1_ generation of *Bemisia tabaci*. Dark red, control (CK) individuals. Gold, individuals treated with the 25% lethal concentration (LC_25_) of chlorogenic acid. Values are presented as the mean ± standard error. *p < 0.05 (Student’s *t*-test).

**Figure 4:**
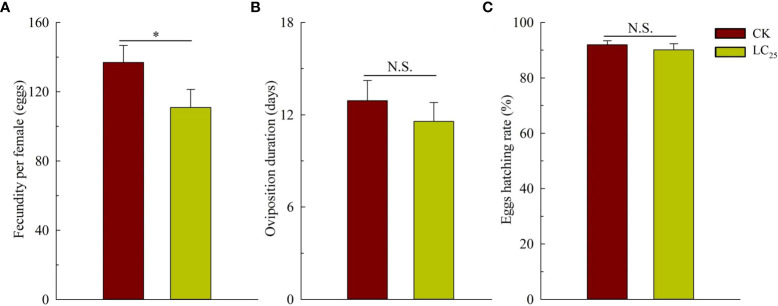
Fecundity **(A)**, oviposition duration **(B)**, and egg hatching rate **(C)** of the F_1_ generation of *Bemisia tabaci*. Dark red, control (CK) individuals. Gold, individuals treated with the 25% lethal concentration (LC_25_) of chlorogenic acid. Values are presented as the mean ± standard error. *p < 0.05 and n.s. indicates not significant (p > 0.05) (Student’s t-test).

In the work of cross-resistance, we assessed the lethality of various concentrations of chlorogenic acid in the MED-S strain, and as shown in the **Table 2**, LC_50_ value was 0.888 with the Slope ± SE was 1.192 ± 0.133, and *X* ^2^ (*df*) was 0.827 (3). Based on the calculation, the value of LC_25_ was 0.241 mg/L, and it was used for further evaluation of the sublethal effects of chlorogenic acid on *B. tabaci* development and reproduction. The results showed that treatment of *B. tabaci* adults with the LC_25_ dose significantly decreased the survival rates of F_1_ progeny in the neonate to pseudopupae stage and in the pseudopupae to adult stage (**Figure 3A**). The F_1_ progeny of treated insects also showed greatly extended durations of these two developmental stages (**Figure 3B**). Moreover, treatment with the LC_25_ dose greatly decreased fecundity in female whiteflies; treated females produced 110.93 ± 11.40 eggs each, compared to the 136.87 ± 9.89 eggs produced by females in the control group (**Figure 4A**). However, there were no significant differences in the duration of oviposition (**Figure 4B**), 11.57 ± 1.23 days in the treatment group vs. 12.92 ± 1.31 days in the control group, and also in egg hatchability (**Figure 4C**), 90.11 ± 2.18% in the treatment group vs. 91.93 ± 1.46% in the control group.

## 4 Discussion

Plant specialized metabolites are considered important candidate compounds in development of botanical insecticides as alternatives to conventional chemical pesticides. However, there is still a dearth of information regarding the bioactivity of botanical toxins against whiteflies. In the present study, we found that the specialized metabolite chlorogenic acid showed higher toxicity than 11 popular commercial insecticides against *B. tabaci* adults (laboratory strain MED-S). Using *B. tabaci* samples collected from the field, we then established the baseline susceptibility of 12 separate populations to chlorogenic acid and assessed cross-resistance to the pesticides abamectin, afidopyropen, cyantraniliprole, and flupyradifurone. All of the tested field-sampled populations were highly susceptible to chlorogenic acid, and chlorogenic acid showed little cross-resistance with abamectin, afidopyropen, cyantraniliprole, and flupyradifurone. Although there have been few previous investigations into the toxicity of chlorogenic acid against *B. tabaci*, chlorogenic acid reportedly exerts excellent lethal effects against various insect pests such as *M. separata*, *Hyphantria cunea*, and *Lymantria dispar* (Wang et al., 2014; Pan et al., 2020; Lin et al., 2022). These characteristics make chlorogenic acid a promising candidate botanical pesticide for use as a more environment-friendly option in field applications compared to synthetic insecticides.

Previous studies of *B. tabaci* have indicated that metabolic resistance to popular chemical agents involves increased activity of P450 enzymes and up-regulation of P450 genes (Zhou et al., 2020; Wang et al., 2020a; Wang et al., 2020c). Here, we selected 12 candidates of detoxifying P450 genes and measured expression levels after chlorogenic acid treatment. After treatment with the LC_50_ dose for 96 h, P450 enzyme activity was greatly induced; furthermore, five P450 genes were significantly up-regulated and one was down-regulated in comparison with the untreated control group. We thus concluded that those six genes (*CYP6CX3*, *CYP6CX4*, *CYP6DW3*, *CYP4C64*, *CYP4G68*, and *CYP6DZ4*) were involved in detoxifying chlorogenic acid. P450 genes have crucial detoxification functions in many insects; pesticide resistance relies primarily on xenobiotic metabolism *via* cytochrome (CY) P450s (Lu et al., 2020; Nauen et al., 2021). Similarly, phytochemicals can induce changes in the expression levels of detoxification-related P450 genes. For example, two P450 genes, *CYP4M14* and *CYP4L13*, are significantly up-regulated in *Spodoptera frugiperda* larvae after exposure to flavonoids and nicotine (Wang et al., 2022c). It was recently reported that chlorogenic acid can induce P450 enzyme activity and that the genes *CYP6B7-like*, *CYP321A7*, and *CYP6B6* are responsible for chlorogenic acid detoxification in *M. separata* (Lin et al., 2022). We therefore speculate that these genes, some of which were significantly up-regulated in *B. tabaci* after chlorogenic acid treatment, may be detoxification genes; furthermore, the insecticidal effects of chlorogenic acid against *B. tabaci* may be due to P450 gene suppression, preventing detoxification and thus resulting in insect death.

Plant-derived pesticides not only have lethal capacity, but also affect insect physiological functions such as behavior, viability, reproduction, and development at sub-lethal concentrations (Toledo et al., 2019; Piri et al., 2020). For example, treatment of *B. tabaci* with the LC_25_ dose of the phytochemical compound β-asarone can prolong the developmental duration, decrease viability, and significantly reduce the rate of reproduction (Wang et al., 2022a).We here found that treatment with the LC_25_ dose of chlorogenic acid had several effects on *B. tabaci*: it prolonged the duration of two developmental stages; decreased survival rates of nymphs, pseudopupae, and adults; and significantly decreased female fecundity. These results were consistent with those of previous publications, which have indicated that the duration of *M. separate* larval growth is significantly prolonged after treatment with the LC_20_ dose of chlorogenic acid (Lin et al., 2022). Moreover, in *Helicoverpa zea*, the developmental duration can be prolonged by exposure to caffeic acid and chlorogenic acid (Summers and Felton, 1994). These previous findings combined with the results of the present study indicate that chlorogenic acid can extend the duration of insect developmental stages, decrease rates of pupation and eclosion, and alter the sex ratio of populations and the fecundity of females; chlorogenic acid thus negatively affects development and reproduction of multiple insect pests.

In conclusion, we found that chlorogenic acid displays excellent lethal effect on *B. tabaci* in the both lab-rear strain and field-collected populations. No cross-resistance to four popular insecticides, and five P450 genes that may be involved in the detoxification process was identified in the work. Moreover, it is important to clarify the sublethal effects of a pesticidal agent as part of an overall assessment of its suitability for field applications. The present study reveals novel insights into the sublethal effects of chlorogenic acid on whiteflies, promoting efficacious use of this compound and contributing to decreased whitefly management costs and crop yield losses due to herbivory.

## Data availability statement

The original contributions presented in the study are included in the article/**Supplementary Material**. Further inquiries can be directed to the corresponding authors.

## Author contributions

Conceptualization, RW, JW and CL. Methodology, RW and QZ. Software, QZ. Validation, CQ. Formal analysis, QW. Investigation, RW, QZ and JW. Resources, CQ. Data curation, QW. Writing—Original draft preparation, RW. Writing—review and editing, RW, JW and CL. Visualization, RW. Supervision, RW, JW and CL. Project administration, RW and CL. Funding acquisition, RW. All authors contributed to the article and approved the submitted version.
