# Supplementary material for: Toxicity, baseline of susceptibility, detoxifying mechanism and sublethal effects of chlorogenic acid, a potential botanical insecticide, on Bemisia tabaci
Source: Front Plant Sci. 2023 Feb 22;14:1150853. doi: 10.3389/fpls.2023.1150853 (PMC9992418; doi:10.3389/fpls.2023.1150853)
Supplement: Supplementary file 1 [file DataSheet_1.docx]

**Table S1.** Primers used in quantitative real-time PCR.

| Primer name | Sequence (5’–3’) |
| --- | --- |
| CYP6CX1 | Forward: GTATCGGATTACGCCCTTCACC |
|  | Reverse: GCAGCCAAACTTCACCTTTCG |
| CYP6CX3 | Forward: CGCATTCTTCCAGTTCCTCGAGA |
|  | Reverse: GGCCATAGCATCCTTCGTGACC |
| CYP6CX4 | Forward: TTGACAAACTTGCGGGGAACCTC |
|  | Reverse: CACAGTCTTTCAGCGTCTCGT |
| CYP6CX5 | Forward: GACTTTCCAGCTGCTCAACCC |
|  | Reverse: GTTCCCGCTGAGCTTGTCCA |
| CYP6CM1v1 | Forward: CACTCTTTTGGATTTACTGCACCC |
|  | Reverse: GTGAAGCTGCCTCTTTAATGGC |
| CYP6DW2 | Forward: CGCTGGAAAAACATCCGCAC |
|  | Reverse: TTTGCGTCCAGGTATCCGTT |
| CYP6DW3 | Forward: CTTACGAATTACCGAACTCAC |
|  | Reverse: CGAACTTCTCAGGCTTAGG |
| CYP6DZ4 | Forward: CGTCAAGCACCGAGAATCAA |
|  | Reverse: ATCCTTAGCGTGACCATCCTG |
| CYP6DZ7 | Forward: CGGAACACGCCTGACGAAAG |
|  | Reverse: GTAACGCACATTCTTCCATCTCTG |
| CYP303A1 | Forward: CGCTCCGGTACAATGCTAATCG |
|  | Reverse: CCCATACACCTGTGTTTACCGAAT |
| CYP4C64 | Forward: CCCTCAAACGGTCCTTCCAAC |
|  | Reverse: GTAATTCTGCGTCTTCGTCAACTG |
| CYP4G68 | Forward: GGTGTATCATGGAGACTCT |
|  | Reverse: GCTGGACTTCTTGTTGTAG |
| TUB1α | Forward: CACTGTTGTTCCTGGTGGC |
|  | Reverse: AGTGGACGAAAGCACGCTTG |
| EF1α | Forward: TAGCCTTCGTGCCAATTTCCG |
|  | Reverse: CCTTCAGCATTACCGTCC |

**Table S2.** Susceptibility of chlorogenic acid in field-collected *B. tabaci* populations from China.

| Population | N ^a^ | LC_50_ (95% CL) (mg L^-1^) ^b^ | Slope ± SE | *X*^2^ (df) | RR ^c^ |
| --- | --- | --- | --- | --- | --- |
| MED-S | 622 | 0.962 (0.743 - 1.182) | 1.291 ± 0.135 | 2.147 (3) |  |
| LY | 627 | 1.369 (1.104 - 1.679) | 1.159 ± 0.127 | 1.552 (3) | 1.4 |
| CY | 610 | 2.327 (1.902 - 2.834) | 1.236 ± 0.129 | 2.289 (3) | 2.4 |
| HD | 623 | 1.021 (0.831 - 1.256) | 1.173 ± 0.128 | 1.793 (3) | 1.1 |
| TZ | 636 | 1.924 (1.572 - 2.350) | 1.193 ± 0.127 | 2.026 (3) | 2.0 |
| WQ | 614 | 3.306 (2.601 - 4.063) | 1.178 ± 0.130 | 2.888 (3) | 3.4 |
| JH | 632 | 1.215 (0.982 - 1.459) | 1.347 ± 0.133 | 0.565 (3) | 1.3 |
| ZJK | 621 | 2.821 (2.117 - 3.739) | 1.470 ± 0.133 | 3.070 (3) | 2.9 |
| BD | 611 | 1.724 (1.364 - 2.082) | 1.497 ± 0.144 | 1.390 (3) | 1.8 |
| ZZ | 616 | 0.723 (0.567 - 0.895) | 1.112 ± 0.128 | 2.174 (3) | 0.8 |
| XZ | 619 | 1.164 (0.895 - 1.446) | 1.133 ± 0.129 | 2.975 (3) | 1.2 |
| JN | 623 | 2.169 (1.599 - 2.756) | 1.039 ± 0.128 | 1.815 (3) | 2.3 |
| TA | 630 | 3.241 (2.444 - 4.100) | 1.006 ± 0.125 | 0.836 (3) | 3.4 |

^a^ Number of insects used. ^b^ CL = confidence limits. ^c^ RR (resistance ratio) = LC_50_ (field-collected population)/LC_50_ (MED-S).
